# A Dynamic Network of Proteins Facilitate Cell Envelope Biogenesis in Gram-Negative Bacteria

**DOI:** 10.3390/ijms222312831

**Published:** 2021-11-27

**Authors:** Chris L. B. Graham, Hector Newman, Francesca N. Gillett, Katie Smart, Nicholas Briggs, Manuel Banzhaf, David I. Roper

**Affiliations:** 1 School of Life Sciences, University of Warwick, Coventry CV4 7AL, UK; Chris.graham@warwick.ac.uk (C.L.B.G.); H.Newman@warwick.ac.uk (H.N.); F.Gillett@warwick.ac.uk (F.N.G.); katiesmart93@gmail.com (K.S.); N.Briggs@warwick.ac.uk (N.B.); 2 School of Biosciences, University of Birmingham, Birmingham B15 2TT, UK; m.banzhaf@bham.ac.uk

**Keywords:** peptidoglycan, interactions, *Escherichia coli*, outer membrane, envelope, network, protein-protein, seds, complexes, dynamic, gram-negative, cell division, cytoskeleton

## Abstract

Bacteria must maintain the ability to modify and repair the peptidoglycan layer without jeopardising its essential functions in cell shape, cellular integrity and intermolecular interactions. A range of new experimental techniques is bringing an advanced understanding of how bacteria regulate and achieve peptidoglycan synthesis, particularly in respect of the central role played by complexes of Sporulation, Elongation or Division (SEDs) and class B penicillin-binding proteins required for cell division, growth and shape. In this review we highlight relationships implicated by a bioinformatic approach between the outer membrane, cytoskeletal components, periplasmic control proteins, and cell elongation/division proteins to provide further perspective on the interactions of these cell division, growth and shape complexes. We detail the network of protein interactions that assist in the formation of peptidoglycan and highlight the increasingly dynamic and connected set of protein machinery and macrostructures that assist in creating the cell envelope layers in Gram-negative bacteria.

## 1. Peptidoglycan in Gram-Negatives

**Figure 1:**
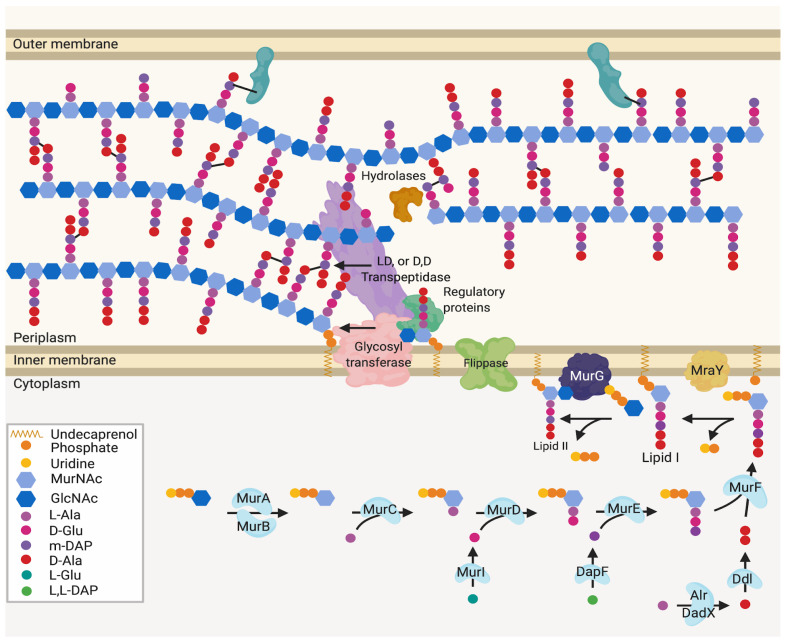
Generalised peptidoglycan synthesis and insertion pathway. Lipid II is the peptidoglycan building block precursor. This precursor is synthesised in the cytoplasm by sequential enzymatic steps then attached to undecaprenyl phosphate in the inner membrane [2,4]. The newly formed Lipid II is then flipped across the inner membrane and polymerised into glycan chains by the glycosyltransferase (GT) action of class A bifunctional penicillin-binding proteins (PBPs), Sporulation, Elongation or Division proteins (SEDS) in complex with class B monofunctional PBPs or monofunctional glycosyltransferases [5].

Peptidoglycan plays a vital role in the maintenance of cell envelope integrity in bacteria generally, and in Gram-Negative bacteria it acts as a stabilising structure that is attached to both the inner and outer membrane lipid bilayers [1]. The peptidoglycan layer is formed of a repeating beta-1–4-linked *N*-acetylmuramic acid N-acetylglucosamine disaccharide glycan polymer (MurNAc-GlcNAc) with crosslinked peptide side chains. The peptide side chains of each of these polymers can extend from the MurNac sugar and crosslink to create a macroscopic mesh-like structure (Figure 1) [2]. The cell constantly modifies this mesh-like macromolecule with a set of hydrolases to break the bonds involved and transferases to form new polymers allowing for cell expansion, shape changes, and septation. A recent review covers these modifications and the proteins involved in detail [3].

## 2. Cell Wall Modifying Enzymes and Complexes Have Altered Localisation during Growth Which Is Essential for Specialised Peptidoglycan Biosynthesis

**Figure 2:**
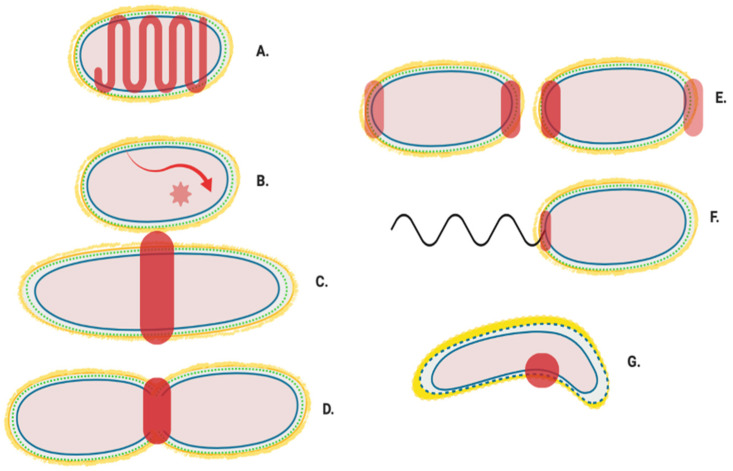
Generalised localisation of peptidoglycan modifying proteins. Localisation regions of known and potential peptidoglycan modifying enzymes in Gram-negative bacteria. Localisation sites are highlighted in red. (**A**) Helical and MreB associated Elongasome; (**B**) Free diffusion (unlocalised) [11]; (**C**) Pre-septal machinery; (**D**) Division machinery; (**E**) Post septal polar machinery and polar growth [13,14,15]; (**F**) Flagella peptidoglycan modification machinery [16], and (**G**) Shape determining pinpoint/seam [17,18].

The location of the enzymes required for the synthesis of peptidoglycan and its later modification (Figure 1) can vary, dependent upon cellular events and conditions [6,7]. The proteins and complexes involved are also dynamic, as many of their locations have been shown to change during the cell cycle. Studies using fluorescent gene fusions within the chromosome and peptidoglycan protein tracking approaches [8,9,10] now provide indications of coordinated peptidoglycan protein complex movement during the cell cycle [11,12] (Figure 2A–C). Localisation of these complexes presumably ensures that peptidoglycan is synthesized at particular regions for either overall growth or highly specialised growth situations such as cell division (Figure 2D); cell curvature; (Figure 2G) polar growth and maintenance (Figure 2E); as well as flagella associated regions (Figure 2F).

## 3. Regulation of Peptidoglycan Modifying Enzymes by Their Interacting Partners

**Figure 3:**
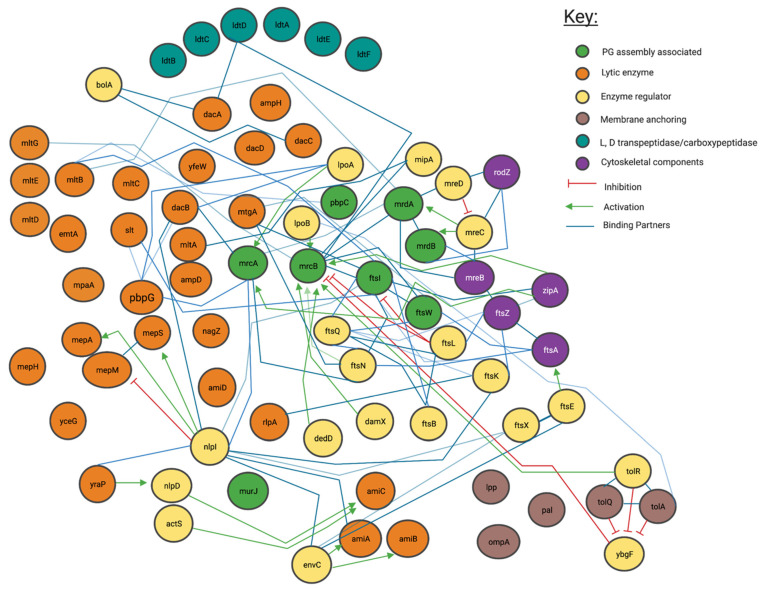
Interaction network of peptidoglycan modifying enzymes and their partners from Literature inspection. Interaction map of peptidoglycan-associated proteins sorted by enzymatic action. Network structure determined by STRING, with manual addition of interactions through literature associated with each protein. Reference matrix available in Appendix A.

To achieve such diversity in the form and location of peptidoglycan, its synthesis and subsequent modification must be highly coordinated. Peptidoglycan is a complex three-dimensional molecule with architecture and chemistry which is dependent on host species and environmental localisation [19,20]. Cells also respond (via modified synthetic pathways) to antibiotic challenge and changing osmotic conditions by changes in their cell wall architecture and peptidoglycan biochemistry [21,22] (Figure 3).

The specialisation of peptidoglycan has been postulated to be driven by pathways that are regulated by local enzyme concentrations and protein: protein interactions. Integral peptidoglycan synthesis complexes such as RodA-PBP2 and FtsW-PBP3 have been shown to have non-enzymatic regulatory partners such MreC/MreD [23] and FtsN/L/Q respectively [24]. In addition enzymatic regulatory pairs exist e.g., PBP1A-PBP2 and PBP1b-PBP3. These networks of peptidoglycan synthesizing enzymes and regulatory proteins is still not understood either structurally or functionally.

## 4. Method Used to Visualise PG Synthesis Networks for This Meta-Review

To visualise the interactions of the genes and proteins relevant to peptidoglycan synthesis and allow a full informative meta-analysis, we have performed a network analysis of relevant genes using contemporary bioinformatic approaches [25].

### 4.1. Genetic and Protein Interactions Confirmed by the Literature

**Figure 4:**
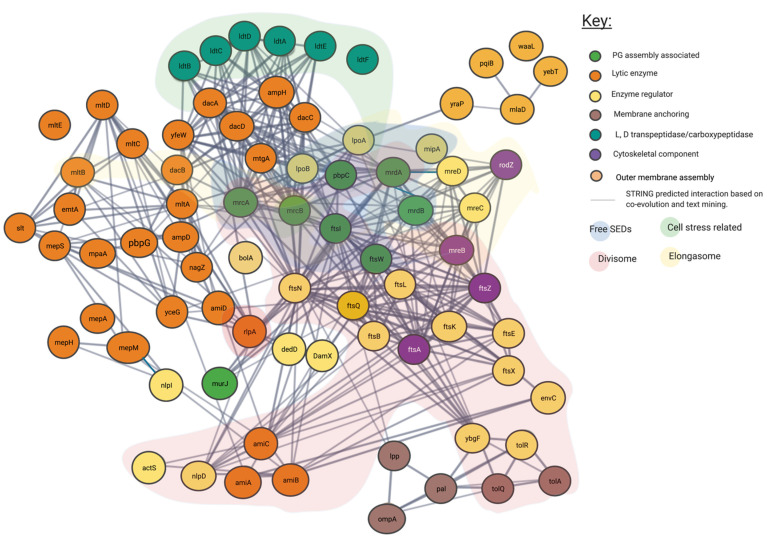
Predicted interaction network of peptidoglycan associated proteins using STRING. Interaction map of peptidoglycan associated proteins, sorted by enzymatic action. Network structure determined by STRING. SEDs complex interaction type is indicated by area colouring and arrows. Interactive network link: https://version-11-5.string-db.org/cgi/network?taskId=bIzLkBRoqjLb&sessionId=bBi0rwtoih3p (Last accessed on 4 November 2021).

Peptidoglycan modifying and related genes, as listed in cell division, peptidoglycan biosynthesis, and peptidoglycan related papers centred on *E. coli* were collated to create a peptidoglycan relevant gene list [6,24,26]. We submitted this list of genes as a joint submission to the gene data trawling engine “STRING” (STRING) to create an interaction map centred around our listed proteins’ data. Genes that interacted with this initial list, or were not in our initial list and given a combined “STRING” score of ≥0.7 (determined by co-occurrence data among species, gene neighbourhood scores and/or experimental data) [26,27] were then added and this new list was inputted as a meta-submission. Those genes which after meta-submission were found to have compound interaction scores with other listed genes > 0.9 were used in our final literature analysis (Figure 3 and Figure 4). This has created a comprehensive picture of the current literature representing peptidoglycan synthesis and modification in Gram-Negative bacteria (Figure 3).

### 4.2. Proposed Genetic Interactions

In addition to the confirmed interactions uncovered by the literature as shown in Figure 3, we show the predicted network used to create it. In Figure 4 unconfirmed STRING determined interactions of 0.7 or higher are shown. This more expansive and connected network represented in Figure 4 was constructed using a database of known and predicted gene-gene/protein-protein interactions derived from direct (physical) and indirect (functional) genetic associations, along with interactions aggregated from other (primary) databases [26,28]. We have grouped proteins currently linked to specific peptidoglycan assembly machinery and their cellular locations into their respective groups through the layering of background colour. This creates a gene-gene interaction pattern network, contextualised by the large protein assemblies they associate, such as the “divisome” and the “elongasome”, as well as other potential environment dependent complexes.

## 5. Most Peptidoglycan Synthases and Modifiers Are Members of Multiple Local Complexes as Predicted by Genetic Interactions and Confirmed by Literature

Our network analysis (Figure 3, Figure 4 and Figure A3) suggests that the overlapping protein complex localisations of Figure 2 such as those related to cell stress, the “divisome“ and “elongasome” involve some of the same proteins, which are shared among complexes. In particular our analysis of Figure 3, which focuses on known literature-verified interactions, indicates that some proteins interact with multiple complexes that facilitate coordination at the cytoplasmic membrane and outer membrane. These interactions can involve lytic, regulator, anchoring, cytoskeletal, and anabolic enzymes, often acting as partners to the same proteins.

**Figure A1:**
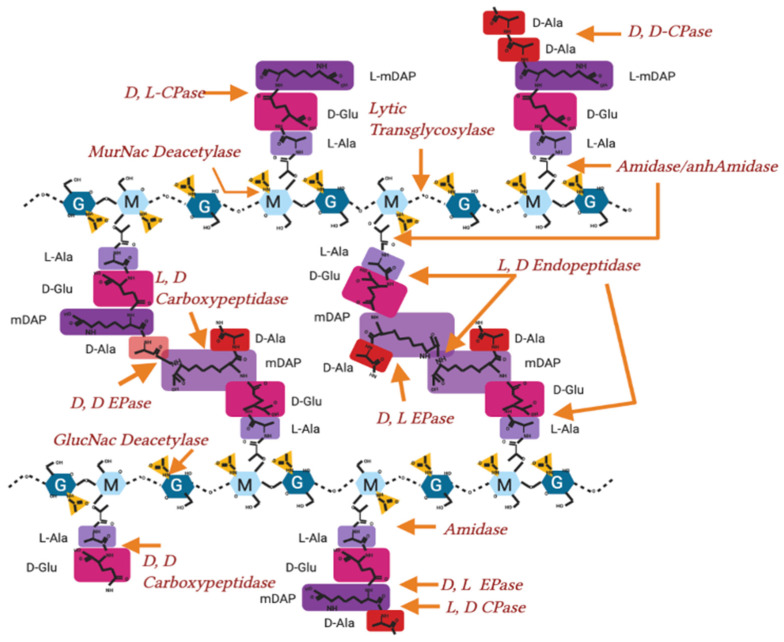
Hydrolase activity on peptidoglycan in Gram-negative bacteria. Peptidoglycan molecular structure, with sites of hydrolysis and enzyme nomenclature labelled. EPase-Endopeptidase, CPase-Carboxypeptidase.

**Figure A2:**
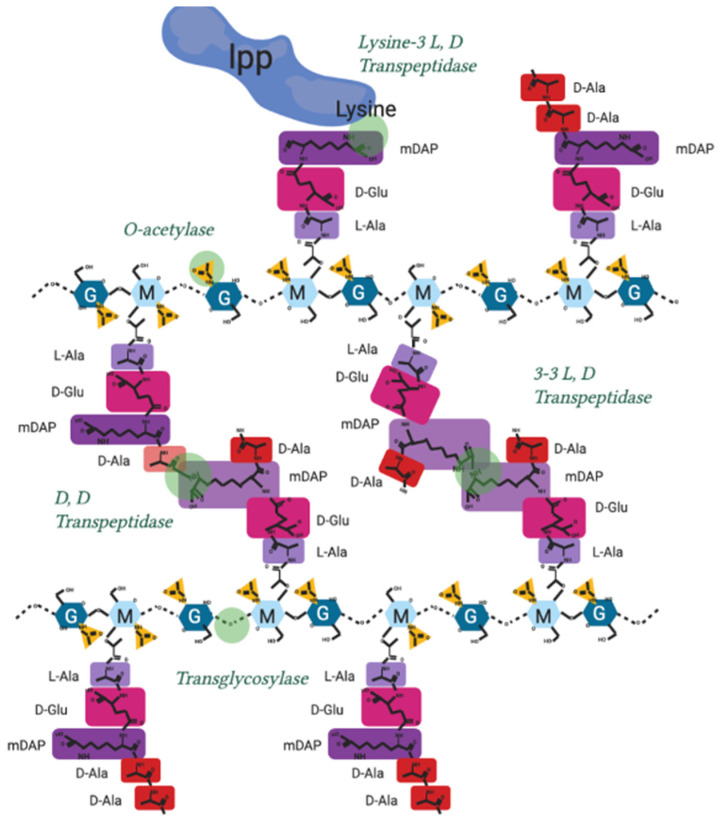
Peptidoglycan crosslinking sites and activity in Gram-negatives. Peptidoglycan molecular structure, with sites of crosslinking actions and enzyme nomenclature labelled.

**Figure A3:**
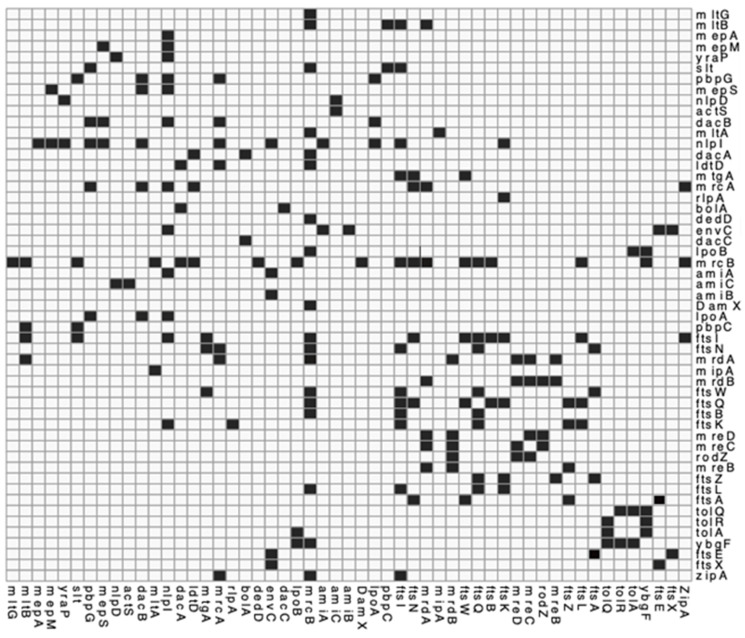
Interaction matrix of PG related proteins. genes or proteins known to interact and confirmed by the literature. Black = confirmed interaction, White = unknown/self/no interaction. Please find attached pg_network.csv file for DOI matrix of confirmed interactions for above.

The central message revealed by the networks suggests that regulation of proteins occurs in complexes, but also through protein exchange and sharing occurs to enable a range of possible additional ensembles. The roles of each protein, and an interaction table for these proteins in the network above are discussed in Appendix A, Table A1, Table A2 and Table A3 as well as Figure A1, Figure A2 and Figure A3. A datafile of the literature in context of this interaction matrix is available (Supplementary Materials).

The networks of literature and genetic interaction in Figure 3 created through the use of an interaction matrix from the literature confirms already widely held theories, that the differences in central peptidoglycan formation units beyond the elongasome and divisome, in addition to other peptidoglycan formation nodes are often based upon changes in accessory proteins and exchange of core proteins and there is rarely a fixed static complex. Figure 4 is derived from a variety of different data sources and shows a complex web of possible interactions. However, all these interactions are unlikely to happen simultaneously in the cell and represent a large spectrum of possible connections. Some of these interaction have been observed experimentally as shown in Figure 3, with proteins such as PBP1b making interactions with a large array of other protein partners, actions highly unlikely to all occur at the same time. This concept exemplifies the observation of several proteins theoretically occurring in multiple protein complexes as indicated by Figure 4. These figures show the overwhelming complexity of the peptidoglycan protein network, as a collection of many complexes.

The pattern of protein re-use and interdependence however is not constant, for example, the monofunctional glycosyltransferase activity of FtsW and RodA have already been shown to be reliant on their partner class B PBP interactions [24,29,30]. These partner dependent glycosyltransferase proteins form a complex with specific class B PBPs such as *mrdA*/PBP2 for RodA and *ftsI*/PBP3 for FtsW (Figure 2) and in doing so produce codependent glycosyltransferase and transpeptidase peptidoglycan machines [10,31,32]; which are regulated by additional cytoskeletal and regulatory proteins. In *E. coli,* simplistically this includes the RodA-PBP2 (*mrdB-mrdA*) complex for elongation and the FtsW-PBP3 (*ftsW-ftsI*) complex for cell division [10]. These complexes act as nodes, (displayed in Figure 3 and Figure 4) creating the basis of the dependent complexes of the elongasomes and divisomes, which can also interact with each another which we will discuss later in this article.

Existing alongside these functionally relevant codependent multimers, are bifunctional transpeptidases and glycosylase class A PBPs such as PBP1B and PBP1a with the codependent multimers can form interaction partnerships. These have also been shown to form their own complexes and act independently of these peptidoglycan machine nodes to modify and synthesis peptidoglycan, introducing further complexity [33].

All the complexes shown, including the Class A PBP centric ones, contain a host of additional regulatory, structural and enzymatically essential proteins that are shared among them, with interactions that span across the network, each interaction determining their specific overall activity dependent on interacting protein concentration, local substrates and their overall lipid environment [10,29,30,31] (Figure 4). Complexes of proteins such as the web of potential interactions shown in Figure 3 and Figure 4 drive cell envelope synthesis. This review attempts to explain these interactions and their importance through stories presented by the current literature and investigates the specific nodes to which they centre.

## 6. Cytoskeletal Proteins Create Nodes of Complex Formation

As shown in Figure 4, some of the peptidoglycan modifying enzymes can be directly linked to cytoskeletal proteins. These cytoskeletal proteins also act as molecular treadmills [32,34,35] (Figure 4). Typically, two treadmills run around the circumference of the Gram-negative cell, one for division and one for elongation made of FtsZ and MreB filaments respectively [34,36]. FtsZ is a cytoskeletal component, that has been shown to localise and move along the cell circumference during cell division in individual strands that make up a macromolecular “Z ring” [37,38]. Soon after the discovery of FtsZ as a division-essential component, it was shown to interact with the peptidoglycan synthesis machinery of FtsW and FtsI/PBP3 and the regulatory proteins FtsQ, B and L [13,14]. Since then it has been associated with many other penicillin-binding proteins including PBP1b [11,12] (Figure 3). It is thought that the polymerization of FtsZ is responsible for the directional movement of the SEDs complexes during division, constriction and septation [36,39]. As a result, this is an important cell division protein. An antagonist of FtsZ polymerisation, viriditoxin causes cell division defects [40]. In Archaea, its analogous FtsZ and often multiple of its paralogues are integral as division orchestrating proteins associated with pseudomurein laydown. These genes cause cell division defects if not genetically present [41]. Similarly, MreB is another cytoskeletal component implicated in cell shape [42] shown to co-localise with the elongasome associated proteins during cell growth. It has been shown to bind the mur ligases which produce the lipid II precursor. The MreB polymerization antagonist A22 too causes cell morphology defects, highlighting the importance of both MreB and FtsZ as shape determining proteins [43].

Though FtsZ and MreB are important for correct cell growth and cell division, the literature has shown some of the peptidoglycan modifying machinery may only transiently attach to FtsZ and MreB treadmills, although it is not always necessary for their function [12,42]. Recent models of transient FtsI-FtsZ interactions by “Brownian-ratcheting” would suggest the peptidoglycan production and modification complexes move in and out of interaction with cytoskeletal components by a transient system of attachment to a cytoskeletal component, followed by protein wandering, allowing the peptidoglycan altering complex speeds to differ dependent on the degree of cytoskeletal attachment [44]. This “Brownian-ratcheting” model hypothesises a zone of active peptidoglycan producing, slower-moving complexes near the faster moving FtsZ rings or MreB filaments that transport inactive complexes in a dynamic equilibrium of interaction with the cytoskeletal nodes [10,36,37].

## 7. The “Elongasome” Is a Collection of Multiple Complexes

Figure 4 indicates all the known interactions of the complex machinery creating peptidoglycan. To understand the general mechanism for peptidoglycan synthesis and modification across species in the context of all steps involved in peptidoglycan creation, a specific example can be called upon. One of the core peptidoglycan biosynthesis and modifying complexes is the “elongasome”; a biosynthetic complex of peptidoglycan manufacturing machinery spanning the periplasm and inner membrane, used during cell growth.

**Figure 5:**
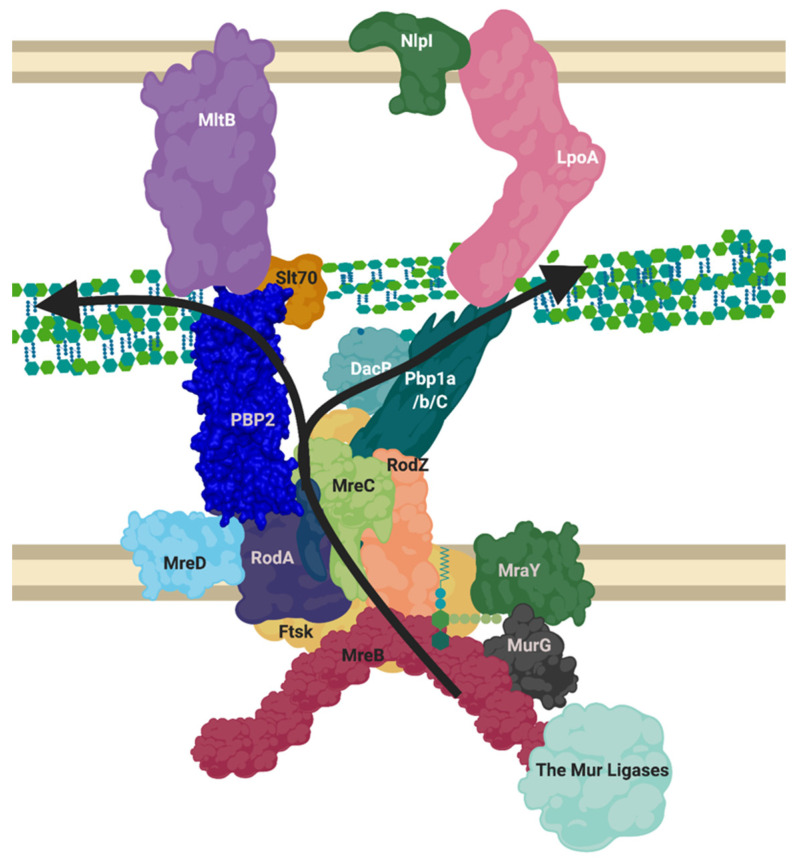
Proposed layout of the RodA orientated elongasome complex. A sketch of peptidoglycan insertion by a proposed formation of the elongasome complex. MreB and C sequester enzymes to the elongasome complex, including RodZ. MurG transforms Lipid I to Lipid II, MurJ/FtsW/RodA flip this into the periplasm. The PBP1a/b/c and/or PBP2-RodA complexes transglycosylate the lipid II into the nascent strand. DacB may remove the terminal D-Alanine from pentapeptide and a transpeptidation reaction occurs through catalysis from transpeptidases PBP2 or PBP1a/b/C. Lytic transglycosylases MltB or Slt70 open new nascent strands for modification as machinery moves forward. LpoA/B along with other regulatory mechanisms listed in Table A3 control PBP activities. This diagram disregards the dynamic nature of the SEDS complexes and MreB ratcheting. Figure created in BioRender.

The elongasome complex (Figure 5) contains the components of generalised peptidoglycan synthesis (Figure 1), but we postulate as have others, based on our bioinformatic analysis and the literature (Figure 3), that the complex also contains class C PBP D, D-carboxypeptidases and lytic transglycosylases to modify peptidoglycan structure and prime it for attachment with new peptidoglycan [1,45]. This model contains the core monofunctional class B transpeptidase PBP2 which inserts its single transmembrane helix into the seven transmembrane helices of RodA, activating it as a glycosyltransferase [46]. This central peptidoglycan biosynthetic capability of the core of RodA-PBP2 is then augmented by the bifunctional Class A PBP, PBP1a also, which associates with the core complex [47].

The scaffolding and regulatory proteins of this elongasome, RodZ and MreC respectively, both interact with cytoplasmic MreB, as well as binding the transpeptidase PBP2 [48]. It has also been shown that MreC, anchored in the membrane with a single transmembrane helix, regulates the crosslinking transpeptidase activity of PBP2, and transglycosylation activity of RodA via interaction of its own periplasmic globular domain with the pedestal domain of PBP2. MreC may also have a role in binding to PBP1a among other components, this is especially interesting as a recent paper shows that *P. aeruginosa* MreC forms large self-storage filaments within the periplasm to likely regulate MreC concentration in the membrane [48,49]. The integral membrane protein MreD has been shown to act as a coordinating partner to MreC in its interaction with RodA and PBP2, with an antagonising effect, however the interaction interface and the regulatory mechanism they perform itself is not yet known. Ribosomal studies suggest MreD levels are half that of MreC, indicating MreC’s storage filaments are likely integral to proper regulation of this system among other possibilities [23].

The elongasome is transiently linked to the cytoplasmic MreB cytoskeleton of Gram-negative bacterial cells [14] (Figure 5). The “Brownian-ratcheting” mechanism of FtsZ (see above) could also apply to MreB interaction, considering the similarity of FtsZ and MreB as cytoskeletal protein homologues. This would suggest that the elongasome complex may instead move in and out of interaction with MreB, rather than remaining always associated [44]. This model would agree with the RodA-PBP2 complex moving along the cell circumferentially alongside MreB bi-modally active and inactive, at different speeds and with alternative partners [11].

Beyond the cytoskeletal interactors, regulation of this elongation apparatus has been shown to require the outer membrane regulatory lipoproteins LpoA and LpoB [25] (Figure 3). LpoA and LpoB span the periplasm to make contact with PBP1a [50] and PBP1b respectively and form synthetically lethal pairings upon genetic deletion, underlying their essential regulatory role [25]. LpoA stimulates the transpeptidase activity of PBP1a specifically, this turn upregulates PBP1a’s glycosyltransferase activity and peptidoglycan production [51] and by contrast, LpoB has been shown to stimulate both PBP1b transglycosylase and transpeptidase activity [52]. Recent analysis of the kinetics of the related PBP1b-LpoB pairing required for cell division, shows that LpoB is an effective on/off kinetic switch for peptidoglycan transpeptidation by PBP1b [25,52]. Therefore, the elongasome contains multiple overlapping and seeming duplicate activities and interactions, but this almost certainly belies the complex network of interactions required for optimal peptidoglycan biosynthesis. One interpretation of this complexity is that the central RodA-PBP2 complex is required for the production of a peptidoglycan scaffold for elongation which the PBP1a-LpoA pairing (connecting inner membrane based synthesis with outer membrane control) then overlays with additional glycan stands and crosslinks, required to produce a complete layered structure [53].

## 8. NlpI Acts as a Facilitator of PBP Nucleation and Complex Interaction

The literature has shown peptidoglycan associated enzymes interact with a great deal of enzymatically inactive structural proteins (Figure 3). A recent paper, has shown there to be an outer membrane protein with a large number of protein-protein interactions, dominating our interaction networks called NlpI [54]. It is postulated to act as a scaffold for peptidoglycan associated proteins and is required for their formation and control. Microscale thermophoresis, pull-down and bacterial two-hybrid studies have shown that NlpI can form trimeric complexes with PBPs, for example, MepS-NlpI-DacA, MepS-NlpI-PBP7 and LpoA/PBP1a/NlpI among many others [54]. NlpI regulates a set of peptidoglycan hydrolases, as well as being able to form a trimer with PBP1A and LpoA. Its absence leads to increased vesicle creation [55] suggesting its importance to the cell envelope. Banzhaf et al. concluded NlpI may facilitate the interaction and/or change the composition of the peptidoglycan editing complexes, controlling the potentially harmful hydrolases and facilitating regulation of other proteins [54].

**Figure 6:**
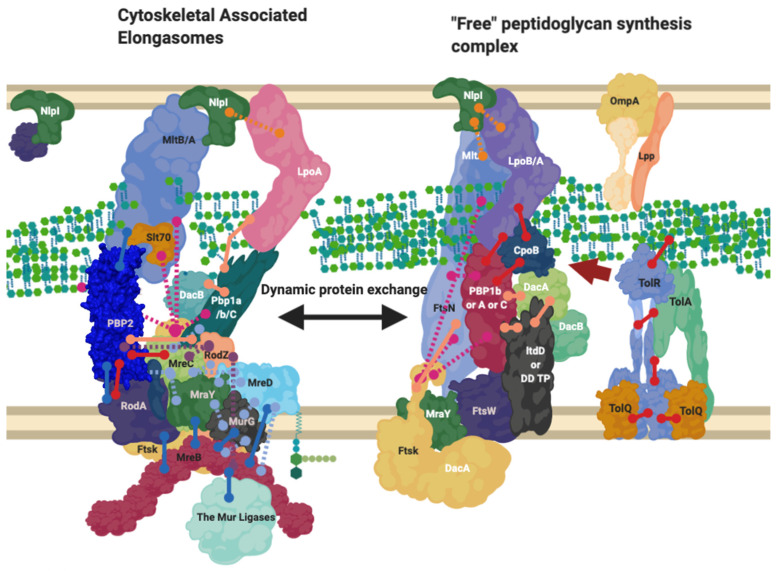
The Elongasome complex interactions. Interactions of the elongasome proteins. The elongasome is a series of complexes that are either attached to or not attached to the MreB filament. Localisation-dependent studies have shown the enzymes involved co-localise at the MreB filament [9,10], and each interaction is cited. This diagram is one of many possible configurations based on the current data, with the incorporation of the flippase not mentioned. Proteins are shared between complexes. Diagram rendered using BioRender. Connections: Purple [57], Red [9,23,31], Cyan [53], Orange [54], Pink [58], Peach [9,29,47,50,59,60], Blue [10,32,61,62,63].

**Figure 7:**
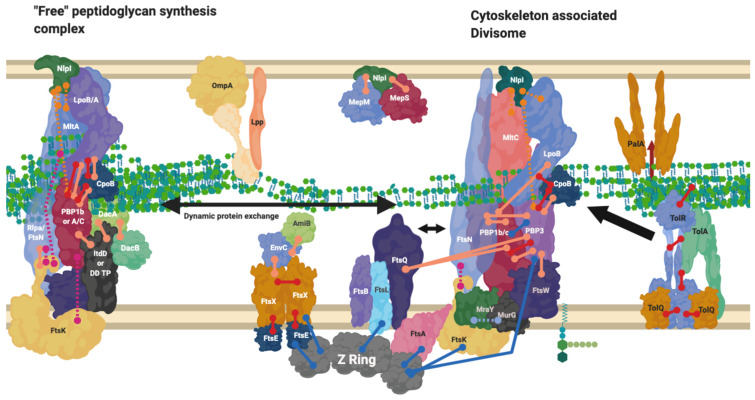
Divisome interaction network at the mature divisome. A diagram representing the interactions of the divisome proteins. Certain proteins within the divisome interact directly with the FtsZ filament. Localisation dependent studies have shown the enzymes co-localise at the FtsZ filament or in the pre-septal region (PIPs). Models suggest interactions are only brief [44]. This diagram is one of many possible configurations based on the current data. Diagram rendered using BioRender. Connections: Purple [57], Red [9,23,31] Cyan [53] Orange [54], Pink [58], Peach [9,29,47,50,59,60], Blue [10,32,61,62,63].

NlpI’s dispersal around the cell indicates it is likely involved in many of the complexes responsible for creating peptidoglycan, including the divisome and elongasome, and possible intermediary complexes that likely exist between those in turn (Figure 6) [56]. As a result, its abundance across the entirety of the cell and regulatory ability suggests it may be part of the system of dynamic protein complex formation this review focuses upon (Figure 3, Figure 4, Figure 6 and Figure 7) and is thus worth noting, however, its role beyond this is not well known [56].

## 9. The Divisome Is a Series of Complexes Controlled by Cytoplasmic Events

The reasoning behind the complex series of interactions in Figure 3 and Figure 4 can be more fully understood in the context of the cell cycle, as not all interactions must occur simultaneously, but rather on a cell stage basis. The divisome is responsible for the division of cells, it is a peptidoglycan modifying complex that has been studied for some time and exists as a series of complex protein-protein interactions (Figure 3), but these have been shown to occur at intermediate stages and be dynamic [3]. The divisome’s function is similar to that of the elongasome’s with analogous flippase, transglycosylase and transpeptidase partners, which are dependent upon a cascade of interactions [9,13,14,55,64]. The divisome proteins that modify peptidoglycan such as PBP3 and PBP1b are not always present with the divisomes central transglycosylase FtsW, as they change their cellular localisation dependent on the cell cycle and by their interactors (Figure 7) [9]. There is a higher concentration of peptidoglycan synthesis and hydrolysis enzymes at the septa during cell division, in a series of stages and cascades, suggesting a dynamic system much like the elongasome system, where protein composition changes over time as need and function changes [63].

Networks of interaction presented in Figure 3, Figure 4 and Figure 7 make clear the abundance of protein interactions possible. Division must account for osmotic conditions, cytoplasmic events, antibiotic challenge, and periplasmic protein complexes, whilst also maintaining the stability of cell envelope layers to prevent cell lysis, and finally allow septation. Recent reviews on the cascade of proteins and steps involved make the changes in the division complex over time clear [12,29,39].

## 10. Proteins Interchange between Complexes, and Complexes Interact

Throughout this review, it is mentioned that proteins can exist in more than one complex (Figure 4). Despite the notion that PBP2 and PBP1a are normally associated with the elongasomes as discussed above, they have also been found at the division site. A hypothesis involving a balance between the elongasome and the free-floating or unbound elongasome was investigated in the Gram-Positive, *Bacillus subtilis*, which found that PBP1a’s homologue can move independently of MreC homologue or RodZ in the cell [64,65]. In the same study, the quantity of MreC and PBP1a also determined the cell width, suggesting this balance of two systems: one elongasome free of cytoskeletal proteins and RodZ, which can diffuse across the circumference of the cell to allow radial expansion, and one which interacts transiently with the cytoskeleton dominates and dominates the elongation process, in addition to morphology determination in *B. subtilis*, which could also be indicative of Gram-Negative systems (Figure 4 and Figure 6) [64,65].

PBP1b/*mrcB* is a bifunctional glycosyltransferase and transpeptidase enzyme that interacts and plays part in the regulation of the divisome. It dominates Figure 3 and Figure 4 as a node with high levels of interaction, beyond which is reasonable to exist at any one time simultaneously [9]. In contrast to PBP1a/*mrcA*, PBP1b/*mrcB* has been postulated to have division complex roles as well as a wandering role [10]. PBP1b and its partner activator LpoB are essential to peptidoglycan rebuilding in peptidoglycan-deficient spheroplasts of *E*. *coli* [33]. Their essentiality outside of division processes to create new peptidoglycan in spheroplasts suggests that PBP1b must play a major role in the creation of new peptidoglycan, which in wildtype cells (*E. coli*) is carried out 70–80% by the bifunctional PBPs such as PBP1b and PBP1a which have roles in the elongasome and divisome [10]. This dependence suggests either; the cytoskeleton-bound or free “elongasome” for cell growth including PBP2 and RodA involves PBP1b more than just transiently, or that more than the static model of the elongation machinery RodA-PBP2 exists and PBP1b has a separate role (Figure 6).

A “free” diffused PBP1b, and PBP2 have been observed independent of MreB/FtsZ systems by fluorescent localisation [10,11]. The interactions shown in Figure 3 and Figure 4 suggest many possible complexes, that vary in their composition, position, and association with the cytoskeleton by PBP1b, PBP1a and PBP2.

The complexes that contain these interactive proteins may also interact. The elongation and division machinery share common protein components and interactors (Figure 3 and Figure 4) with the elongation machinery associated “PBP2” even transiently localising to the Z-ring during cell division [63]. PBP2, a protein known to be integral to the elongation machine, has also been shown to interact with PBP3s division related role (*ftsI*), with PBP2 knockout studies revealing division defects. In addition to division and elongation related localisation, the peptidoglycan synthase proteins PBP2, PBP1a, PBP1b and FtsW have also been shown to localise diffusely around the cell, moving independently to the cytoskeletal-associated elongation and division complexes [9,10,12].

During the midstage of division, MreB and FtsZ appear to co-localise at the Z-ring whilst treadmilling [63]. It has been postulated that enzyme exchange of these proteins between the divisome and elongasome may occur through an interaction with the cytoskeletal components MreB and FtsZ [13,63]. This would support the “Brownian ratchet” model theory of cytoskeletal protein control, citing a transient interaction rather than permanent interaction of the PG machinery with FtsZ and MreB, allowing for the exchange of proteins between cytoskeletons more easily [44].

PBP1b and PBP2 localise to the septum adjacent to the Z-ring during division, and become delocalised from the septum in an *mreB* knockout strain [13]. Mutation of the FtsZ-interacting residue of MreB similarly delocalises PBP1b and PBP2 from the FtsZ rings, despite successful MreB and Z-ring formation. The unused Z-rings remain as “locked” stripes of unsuccessful division sites, and cells containing these Z-rings stripes become filamentous cells. These “locked” Z-rings fail to incorporate fluorescent single D-amino acid probes such as HADA denoting new peptidoglycan biosynthesis, and thus do not actively synthesise peptidoglycan, while the elongation enzymes along the rest of the cell remain functional and successfully incorporate HADA throughout the rest of the cell [13]. This may be due to the absence of the PBP1b-FtsN interaction which would normally interact transiently with MreB’s PBP2, and transition to divisome interactions to relieve the FtsQLB inhibition of PBP3 [24,29,66], without this MreB-FtsZ transient reaction this inhibition remains in place. The cytoskeletal component amino acid knockouts described above, in conjunction with a known lethal PBP2 knockout phenotype, show the necessity of elongation enzymes such as PBP2 to also be required for division and highlights dynamic interchange between complexes [67].

## 11. Alternate Protein Complexes Exist, Containing 3-3 Crosslinking L, D Transpeptidases as an Alternative to 3-4 Crosslinking PBPs Important for Antibiotic Resistance

Figure 3 and Figure 4 and the literature they represent indicates enzymes not yet confirmed to be integral machineries to be involved in interactions in multiple complexes. There is good evidence that during extended growth, osmotic cell stress and some instances of β-lactam challenge, 3-3 crosslinking increases in the peptidoglycan of many Gram-negative bacteria [68]. This form of crosslinking is catalysed by L,D-transpeptidases (Figure A2). The literature, and genetic predictions indicate these proteins interact with many other PBP related proteins (Figure 3 and Figure 4).

**Figure 8:**
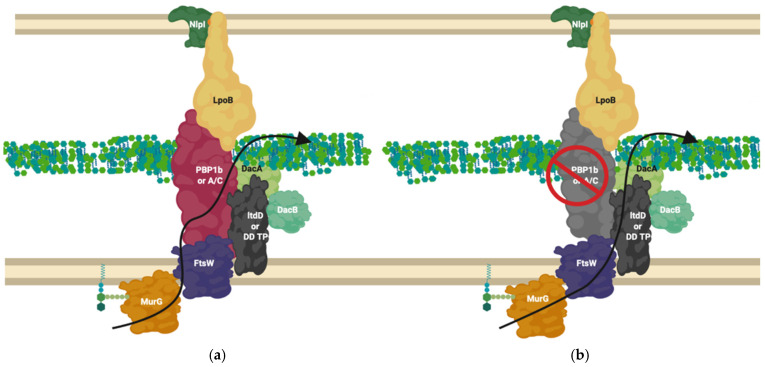
LdtD in complex with PBP1b and DacA. (**a**) PBP1b active complex performs 3-4 crosslinking; (**b**) On β-lactam challenge PBP1b activity is reduced, allowing LtdD, in complex with DacA and DacB, to take over crosslinking and allow cell viability [59,69]. The complex represented above is one of many possible complexes. The flippase is represented by FtsW due to its potential bifunctional role for simplification but likely involves MurJ, this is one of many possible configurations. MurG ligase has been shown to interact with FtsW [60,66].

The L, D-transpeptidase LdtD, has recently been shown to interact with peptidoglycan endopeptidase DacA and bifunctional synthase PBP1b by microscale thermophoresis [59]. Following PBP1b inhibition by β-lactams, LdtD compensates for the loss of 3-4 crosslinking by 3-3 crosslinking, enabling cell survival in the presence of β-lactams [69]. In this situation LdtD could compensate for part of PBP1b’s normal bifunctional role as a transpeptidase (when in complex with the transglycosylase FtsW and PBP3) by replacing the PBP catalysed 3-4 transpeptidase activity with 3-3 crosslinking, using the PBP1b and FtsW transglycosylase glycan chain products as substrates. In this scenario the D, D-carboxypeptidase of DacA, shown to be essential for β-lactam resistance mediated by LdtD [69], modifies the pentapeptide by removal of the terminal amino acid to provide a suitable tetra peptide substrate to LdtD. This is necessary because LdtD requires a tetrapeptide as a substrate [69]. Depending upon the availability of suitable substrates and some environmental conditions, PBP1b will also generate tetrapeptide products which could become substrates for the Ldt’s [52,69]. It is likely this isolated complex is only one of many complexes incorporating the non-canonical peptidoglycan crosslinkers L, D-transpeptidases (Figure 8).

## 12. A “Shapeosome” Complex Synthesises Peptidoglycan in Curved Gram-Negatives

**Figure 9:**
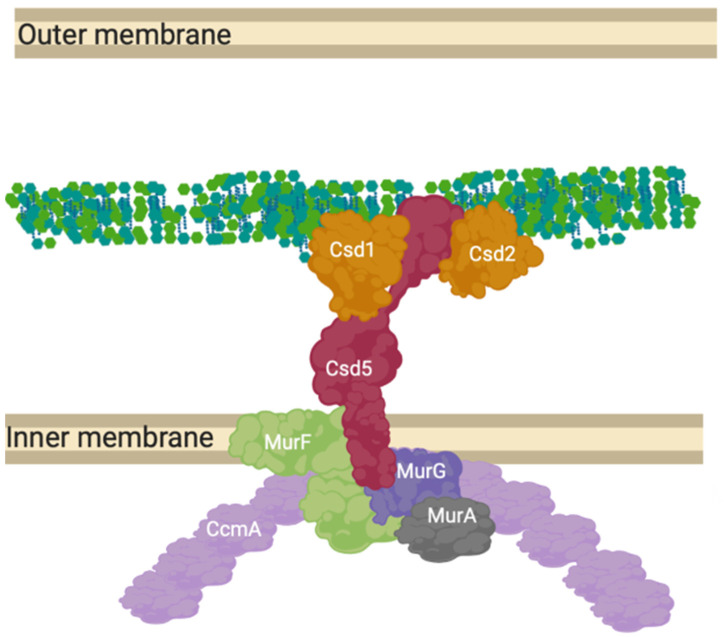
CcmA curvature promoting complex in *Helicobacter pylori.* The prospective anchored “Shapeosome” of *Helicobacter pylori*. The Shapeosome is a non-canonical peptidoglycan synthesis complex that facilitates cell shape of curved bacteria, associated with a cytoskeletal component like the elongasome and divisome. The connection to a cytoskeletal component again suggests a convergent and re-occurring model of shape-determining cell wall modification by complex formation. Csd5 binds peptidoglycan by its SH3 domains and interacts with synthesis related proteins MurG, MurA and MurF, as well as hydrolases Csd1 and 2 [18,72].

Beyond the simple models presented as elongation or division mechanisms, many species of Gram-negative bacteria are more morphologically complex than just rods or spheres [70] and the complex of proteins presented for *E. coli* (Figure 4). These species require the peptidoglycan machinery to be altered compared to these classical exemplar species. *Campylobacter jejuni* and *Helicobacter pylori* have been shown to contain hydrolytic L, D carboxypeptidase proteins essential to cell curvature [71] in addition to a new cytoskeletal component analogue to MreB, CcmA. The most well studied of these systems is *Helicobacter pylori’s* “Shapeosome” and the conserved shape determinant (Csd) protein family. Knockouts of Csd6, CcmA and Csd5 all lead to curvature loss, along with peptidoglycan peptidases Csd1, 2, 3, 4 and 6 [18,72] (Figure 9).

## 13. Unrelated Cell Envelope Proteins must Affect Peptidoglycan-Membrane Linkage

Whilst peptidoglycan is generally regarded as being composed of intramolecular crosslinks [2], in some organisms, L, D transpeptidase enzymes (LDTPs) can catalyse the formation of intermolecular cross links used to attach it to other macromolecular structures [73]. It was shown in the 1970s that at least one-third of Braun’s lipoprotein (Lpp), an outer membrane protein, is bound to peptidoglycan [74] (Figure 1). In Figure 3 and Figure 4, an abundance of outer membrane interactors and regulators, are shown to be part of elongation, and division complexes. It is has been known for some time that in species with and without Lpp, the outer membrane protein OmpA interacts non-covalently with peptidoglycan, but more recently, the literature has shown multiple OMPs across species to be connected covalently to the peptidoglycan by LDTPs [75]. Specifically for example in *C. burnetii* the L, D-transpeptidase ldt2 is required for covalent attachment of OMPs BbpA and BbpB to peptidoglycan [76]. This creates a static covalent link between the outer membrane, peptidoglycan and inner membrane proteins such as Tol machinery across species [62].

Further still, bacterial periplasmic complexes such as pili, transport systems and flagella penetrate through the peptidoglycan layers (Figure 1) and are often able to transport proteins from the inner to outer membrane despite this linkage. This activity requires dynamic pores in the peptidoglycan layer [20,77]. Observed movement of large proteins laterally across these fixed peptidoglycan layers, linked to outer and inner membranes may involve cleaving of the peptidoglycan using lytic transglycosylases to facilitate this movement [16,78,79], in addition to L, D carboxypeptidases, for Lpp release [80]. These protein-peptidoglycan interactions must all be regulated to avoid cell lysis, as shown by multiple periplasmic proteins that ensure the cell envelope remains structurally stable during the cross-periplasmic complex movement. These local changes in cell wall structure due to large complex movements present the cell envelope and peptidoglycan as a more multi-ordered structure than a simple mesh. It must require uncrosslinking and regulatory mechanisms for growth facilitation, and cell envelope stabilisation.

## 14. Review Summary

The peptidoglycan polymer’s complex and essential role to Gram-Negative bacterial cells requires an intricate set of proteins within the periplasm; to maintain its role in response to growth, during division and to ensure a stabilising permeable barrier is maintained in tandem with the inner and outer membrane. The literature has shown this takes place through a series of protein complexes, and this is reaffirmed in predictive genetic and experimental interactions presented (Figure 3 and Figure 4). However, the full picture of experiments, when investigating the roles of each protein have shown that these complex interactions are not static in composition, but are instead part of a web of interactions that allow many variant complexes to be in dynamic equilibrium depending on cell growth stage and need. This model is not yet complete.

Models have been postulated of wandering and cytoskeletal-associated complexes such as the elongasomes and divisomes that create and modify peptidoglycan dependent on growth needs (Figure 6 and Figure 7). Alternative complexes have also been shown to exist for the antibiotic insusceptible L, D-transpeptidase enzymes which can allow crosslinking of peptidoglycan in the absence of the antibiotic susceptible PBPs (Figure 8). These must all occur in the context of structures that cross the periplasm and connect the inner membrane and outer membrane in partnership with other processes [80,81].

This model of large protein complexes evolved to allow for peptidoglycan modification dynamically across a growth cycle and repeats convergently in other species, even among Archaea. Peptidoglycan modification systems, such as the shape determining complex oriented by the cytoskeletal protein CcmA in *Caulobacter* sp. (Figure 9) exist as convergent versions of the *E. coli* MreB and FtsZ based models presented in this review. The cytoskeletal component of some of these dynamic complexes across species, (FtsZ and MreB) treadmill along the circumference of the cell and have been shown to exchange protein partners during their interactions, and cytoskeletal or regulator absence/inhibition leads to growth defects. This evidence among others, shows an exchange of proteins which facilitate a change of complex composition over time by the associated machineries. Sometimes these complex changes are driven by specific cytoplasmic events and cascades, such as those that control the divisome.

However not all modification relies upon these cytoskeletons, as shown by PBP1b wondering motion across the cell [56]. Indeed, a single protein could be required for multiple functions and complexes that exist at once (Figure 3), therefore these multiple protein localisations are in part controlled by affinity to the cytoskeletal proteins or outer membrane proteins anchored such as NlpI. This allows for fine control of complex composition in addition to regulation by protein affinity to local substrate [30].

The peptidoglycan research and anti-microbrial resistance field has come to place importance on specific protein structure, and singular relationships with inhibitory/activator proteins in future antibiotic design. Our meta-analysis has shown the full picture so far likely extends beyond the crystallised complexes and static complexes, revealing a great deal of flexibility, but also indicating the importance of specific nodal proteins in peptidoglycan synthesis. Research into macro-regulation of the complicated cell envelope complexes showcased will be an important step in the creation of new drugs that can overcome known mechanism of antibiotic bypass by protein exchange, but also postulate new methods for peptidoglycan and cell envelope disruption. Viewing these proteins in a systems context will be an important step in combatting resistance to antibiotics in vivo.

## Data Availability

The interaction network of proteins, with DOI citations at each interaction pair can be found in the paper’s supplementary data file. The predicted gene network used for Figure 4 is available at STRING’s website using the permalink: https://version-11-5.string-db.org/cgi/network?taskId=bIzLkBRoqjLb&sessionId=bBi0rwtoih3p (Last accessed on 4 November 2021).

## Supplementary Materials

The following are available online at https://www.mdpi.com/article/10.3390/ijms222312831/s1.

## Appendix A

### Appendix A.1. Mur Ligase Pathway

Fructose-6-phosphate is converted by four successive enzyme activities to uridine 5′-diphosphate-N-acetylglucosamine (UDP-GlcNAc). This is catalysed by GlmS, GlmM and GlmU (bifunctional enzyme) UDP-MurNAc (5′-diphosphate *N*-acetylmuramic acid) is formed from UDP-GlcNAc using Mur ligases MurA and MurB. This results in a sugar moiety ready for pentapeptide addition [78].

A pentapeptide stem is then appended to the D-lactoyl carboxyl group of UDP-MurNAc by sequential addition of peptides by MurC-F: L-Ala (MurC), D-glutamic acid (MurD), meso-diaminopimelic acid (m-DAP) (MurE), dipeptide D-Ala-D-Ala (MurF), with D-Glu and m-DAP being synthesised from their L- or L,L-stereoisomers by MurI and DapF respectively, and D-ala-D-Ala being produced from L-Ala by alanine racemases and D-Ala-D-Ala ligase [78].

The UDP-MurNAc 5P produced by these reactions is then transferred to an undecaprenol, a membrane-spanning lipid, yielding undecaprenyl diphospho MurNAc 5P (Lipid I), in a reaction catalysed by MraY at the inner membrane. Thereafter, MurG transfers a GlcNAc sugar moiety from UDP-GlcNAc to Lipid I, producing undecaprenyl diphospho MurNAc GlcNAc 5P (Lipid II) [66,78].

### Appendix A.2. After Initial Synthesis, Peptidoglycan Is Modified

The modification of peptidoglycan extends far beyond the model in Figure 1, and continues after de novo peptidoglycan insertion, by a series of proteins and protein complexes referred to as PBPs. During synthesis a lytic transglycosylase (Figure A1) separates the existing strands of peptidoglycan to make space for new synthesis by cleaving the links between sugars [79,82], the glycan strand does not continue indefinitely and are typically between 7 and 32 sugars in length [83]. New peptidoglycan must also be attached to the outer membrane by L, D transpeptidase action through linking peptidoglycan to outer membrane proteins like Lpp (Figure A2), roughly once every 100 Å to maintain cell envelope stability [69,80].

During division, the peptidoglycan crosslinks are continually broken and remade to relieve overall cell wall stress and facilitate growth (Figure A1). 3-4 crosslinks being predominant when the cell is not in stress and early in growth, whereas 3-3 crosslinking is found in cells increasingly during the stationary phase, or following antibiotic exposure and osmotic shock [84] (Figure A2).

A shift from 3-4 to 3-3 crosslinking often also occurs during cell growth. Fluorescent D-amino acids (FDAA) have been used to label transpeptidase activity through the ability of PBPs to exchange amino acids, revealing that during stationary phase and growth, the entire peptidoglycan is “lit up” by incorporation of new FDAA indicating new modifications are being made throughout growth [85].

Bacterial cell elongation and division requires the peptidoglycan layer to be constantly modified and cleaved to allow for growth. The attachment of protein partners to the peptidoglycan layer and the peptidoglycan recycling process additionally requires peptidoglycan cleavage. The cleavage and modifications of peptidoglycan varies across species and can be broadly split into two classes of enzymatic action: hydrolase and transferase [12,82] (Figure A1 and Figure A2).

Hydrolases carry out a range of lytic modifications to peptidoglycan, including cleavage of the peptide stem at the glycosidic bond between glycan molecules, and the removal of acetyl groups (a lysozyme resistance factor in pathogenic strains) [85] (Figure A1). The hydrolases so far characterised are dispersed throughout the cell periplasm at the lateral cell wall or the division plane [12].

Hydrolases are controlled by regulatory proteins and each hydrolase has their own distinct role within the cell and the cell’s complexes [86,87] (Table A1).

Peptidoglycan is also polymerised and modified by a series of crosslinking enzymes such as the penicillin-binding proteins (PBPs) or L, D transpeptidases at different points in the bacterial cell cycle as well as after stress events. The modification sites shown in Figure A2 are the points of peptidoglycan crosslinking and attachment known in Gram-negative bacterial species [9,47,88].

Their individual activities are elaborated in Table A2.

**Table A1:** Characterised Hydrolases in Gram-negative bacteria.

Peptidoglycan Degradation/Hydrolases			
Function	Enzymatic Action	Known Genes/Protein	References
D,D Carboxypeptidases	D-Ala D-Ala Cleavage 4-5	dacA, yfeW, dacC, dacD, vanY, ampH, Csd3 *	[69,89,90,91]
MurNac de-Acetylase	Deacetylation of N-acetyl Muramic acid	pgdA	[92]
GlutNac de-acetylase	Deacetylation of N-acetyl Glucosamine		
Amidase	Cleavage of peptide stem from Glycan strand	amiA, amiB, amiC, amiD, ampD, mpaA	[88,90,93,94]
Lytic Transglycocylase	Breaking Glycan strand at GlucNac-MurNac (endo)	Slt, MltA, MltB, MltC, MtD, MltE, PilT, traB, virB1, rlpA, MltG	[16,58,79,82,83,86,95,96]
	Breaking Glycan strand at GlucNac-MurNac(exo)	NagZ	[97]
L, D Carboxy/Endopeptidase	mDAP mDAP cleavage 3-3	mepA	[98]
	mDAP-Lpp Cleavage	YafK/LdtF	[80]
	mDAP D-Ala cleavage 3-4	pgp2 *, csd6 *	[71,91]
	mDAP-D Glucosamine cleave 3-2	csd4 *	[72]
D, D Endopeptidase	Cleavage of D-Ala-mDAP crosslink 3-4	dacB, pbpG, MepS, MepM, PBP7, MepH	[31,99]

* Not present in *Escherichia coli* MG1655 genome, but present in other species.

**Table A2:** Characterised peptidoglycan synthases in Gram-negative bacteria.

Function	Enzymatic Action	Known Genes/Protein (*E. coli*)	References
D, D Transpeptidase and Transglycocylase	Adds lipid II to nascent strand and crosslinks into existing PG	mrcA/PBP1a	[29,100]
		mrcB/PBP1b	[78]
D, D Transpeptidase	Crosslinks nascent strand into existing peptidoglycan	mrdA/PBP2, FtsI/PBP3	[11,101]
Transglycosylase	Adds lipid II to nascent strand	mtgA, rodA, ftsW	[23,102,103]
Flippase	Flips Lipid II to periplasm	murJ	[104]
L, D Transpeptidases	Peptidoglycan Brauns lipoprotein crosslinkers	LdtA/ErfK,	[105]
		YbiS/LdtB	
		Ycfs/LdtC	
	Peptidoglycan 3mDAP-3mDAP crosslinkers	YnhG/LdtE	
		YcbB/LdtD	
O-acetylation	O-acetylates nam	oatA *	[2,92]
		adr	
		pacA	

* Not present in *Escherichia coli* MG1655 genome, but present in other species.

**Table A3:** Peptidoglycan associated and PBP regulatory proteins.

Function	Known Genes/Protein (*E. coli*)	Reference
Moderate class A PBP activity	LpoA, LpoB	[9]
Alter interactor ability	CpoB	[9]
Bind OM with peptidoglycan	OmpA	[106]
Moderate OM linkage with peptidoglycan	lpp	[73]
Periskeletal elongasome component, moderates PBP2 activity	MreC	[23]
Periskeletal elongasome component, moderate PBP2 activity	MreD	
Treadmilling Cytoskeletal elongasome component	MreB	[15]
Elongasome staple component	RodZ	[57]
SPOR domain containing proteins, protein interaction	Rlpa, FtsN, DamX, SpoX	[61,85,107]
Hydrolase binding activity	NlpI	[55]
	ActS	[108]
	NlpD	[109]
	EnvC	[93]
Inner membrane peptidoglycan moderation	TolA, TolR, TolQ, palA, palB	[9,109]
Division moderation and EnvC control	FtsX, FtsE	[110]
Treadmilling cytoskeletal component for divisome	FtsZ	[44,56]
Division moderation	FtsA	[56]
Helicase and PBP interactor	FtsK	[58]
FtsN interactor and division start	FtsB, FtsL, FtsQ	[25]
FtsZ interactor and PIPs mediator	ZipA	[39]
